# Field-derived *Schistosoma mansoni* and *Biomphalaria pfeifferi* in Kenya: a compatible association characterized by lack of strong local adaptation, and presence of some snails able to persistently produce cercariae for over a year

**DOI:** 10.1186/s13071-014-0533-3

**Published:** 2014-11-26

**Authors:** Martin W Mutuku, Celestine K Dweni, Moses Mwangi, Joseph M Kinuthia, Ibrahim N Mwangi, Geoffrey M Maina, Lelo E Agola, Si-Ming Zhang, Rosebella Maranga, Eric S Loker, Gerald M Mkoji

**Affiliations:** Centre for Biotechnology Research and Development, Kenya Medical Research Institute (KEMRI), P.O Box 54840–00200, Nairobi, Kenya; Centre for Public Health Research, KEMRI, P.O Box 20752–00200, Nairobi, Kenya; Department of Zoology, Jomo Kenyatta University of Agriculture and Technology, P.O Box 62000–00200, Nairobi, Kenya; Center for Evolutionary and Theoretical Immunology, Department of Biology, and Parasitology Division, Museum of Southwestern Biology, University of New Mexico, Albuquerque, 87131 U.S.A

**Keywords:** Schistosomiasis, Host-parasite interactions, Compatibility, *Biomphalaria pfeifferi*, *Schistosoma mansoni*, Super-sursvivor snails, Local adaptation, Parasite transmission

## Abstract

**Background:**

*Schistosoma mansoni* is widely distributed in sub-Saharan Africa with *Biomphalaria pfeifferi* being its most widespread and important snail intermediate host. Few studies have examined the compatibility of field-derived *B. pfeifferi* snails with *S. mansoni* miracidia derived from human hosts. We investigated compatibility (as defined by shedding of cercariae following exposure to miracidia) of two isolates of *S. mansoni* from school children from Asao (western Kenya) and Mwea (central Kenya) with *B. pfeifferi* collected directly from Asao stream or the Mwea rice fields.

**Methods:**

We exposed snails from both regions to four different doses of miracidia (1, 5, 10 and 25) from sympatric or allopatric *S. mansoni*, and maintained them in a shaded, screened out-of-doors rearing facility in Kisian, in western Kenya. Both snail survival and the number of snails that became infected were monitored weekly. This was done for 25 weeks post-exposure (PE). Those infected snails which survived beyond this period were monitored until they all died.

**Results:**

Although overall survival of Mwea snails maintained in western Kenya was generally low, both sympatric and allopatric combinations of parasites and snails exhibited high compatibility (approximately 50% at a dose of one miracidium per snail), with an increase in infection rates as the miracidial dose was increased (P < 0.002). Schistosomes were no more compatible with sympatric than allopatric snails, nor were snails less compatible with sympatric than allopatric schistosomes. Snail mortality increased significantly with dose of miracidia (P < 0.05). Approximately 3% of Asao snails exposed to a low dose of sympatric miracidia (1 or 5) continued to shed cercariae for as long as 58 weeks post exposure.

**Conclusions:**

There were no significant local adaptation effects for either schistosomes or snails. Also, the existence of “super-survivor” snails is noteworthy for its implications for current control initiatives that mostly rely on mass drug administration (MDA). Long-term shedders could provide an ongoing source of cercariae to initiate human infections for many months, suggesting care is required in considering how human MDA treatments are timed. Future control programs should incorporate means to eliminate infected snails to complement chemotherapy interventions in controlling schistosomiasis.

**Electronic supplementary material:**

The online version of this article (doi:10.1186/s13071-014-0533-3) contains supplementary material, which is available to authorized users.

## Background

Schistosomiasis remains a major public health problem in the tropical regions of the world, with over 90% of the world’s 207 million cases of human schistosomiasis occurring in sub-Saharan Africa [1]. Among the three major schistosome species causing human schistosomiasis, namely *Schistosoma mansoni*, *S. haematobium* and *S. japonicum*, *S. mansoni* is the most widespread and probably the most important from a public health viewpoint [2,3]. *S. mansoni* has an obligate dependence on freshwater planorbid snails of the genus *Biomphalaria* for its transmission, with *B. pfeifferi* being the most prominent species and most widespread inhabitant of streams and other small water bodies across sub-Saharan Africa [4,5]. Although by far the majority of the world’s cases of *S. mansoni* occur in Africa, most of the experimental work on the snail hosts of *S. mansoni has* been done with *B. glabrata*, a snail restricted to the Neotropics [6].

A key determinant of schistosomiasis transmission success is compatibility of the local snail population to schistosome infection. Compatibility here is defined as the likelihood that exposure to a miracidium or miracidia leads to a cercariae-producing infection. The greater the compatibility, the more snail infections that result from a given level of schistosome egg input into the habitat, the more cercariae that are produced, resulting in increased transmission [7,8]. In Kenya, *B. pfeifferi* is widely distributed, including in the tributaries feeding Lake Victoria, canals in major irrigation schemes in the Kano plains (Western Kenya) or in the Mwea irrigation scheme in central Kenya; it is also found in small impoundments and both seasonal and perennial streams throughout the country, except in the tropical lowland belt along the coast of Kenya [9]. Nonetheless, populations of this species and of the schistosome it transmits can be widely separated by regions of aridity in Kenya. As a consequence, it is possible that *S. mansoni* exhibits a greater degree of compatibility with its local *B. pfeifferi* population than it does with other populations of the same species further removed geographically. Theory predicts that a parasite should be more adapted to sympatric than to allopatric hosts, and that the superior adaptation of a parasite to local hosts should be more pronounced when the host has a discontinuous rather than continuous distribution [10-13]. A number of factors, including high rates of local extinction (such that co-evolutionary associations do not have a chance to develop), high rates of migration of host or parasite populations, or time lags in response, may break down or obscure patterns of local adaptation [12,14]. In Kenya, as assessed by microsatellite analysis, *S. mansoni* from Mwea (central Kenya) and Kisumu (western Kenya) is genetically diverse [15,16]. In addition to being genetically diverse, *S. mansoni* enjoys relatively rapid rates of migration owing to existence of long-lived adult worms in mobile human hosts. By comparison, *B. pfeifferi* is a strong self-fertilizer [17] and its movement is relatively limited owing to its restriction to aquatic habitats. Based on these considerations, *S. mansoni* might be expected to exhibit strong local adaptation to *B. pfeifferi*, as manifested by shorter pre-patent periods, higher compatibility, or higher levels of cercariae production when exposed to sympatric as opposed to allopatric snails. Conversely, *B. pfeifferi* might also be predicted to exhibit local adaptation to schistosomes and consequently show lower compatibility following exposure to sympatric than allopatric schistosomes. These topics have not been addressed in Africa with a reciprocal cross design approach using field-derived snails and parasites not subjected to the biases resulting from prior laboratory propagation; this approach better represents the conditions in natural transmission sites.

Mortality of snails exposed to *S. mansoni* is another key aspect in transmission of schistosomiasis. Again with respect to the issue of local adaptation, it has been suggested that snails exposed to *S. mansoni* from a sympatric source should have a higher survival rate than when the snails are exposed to an allopatric source of *S. mansoni*. This is because selection imposed by local parasites is expected to favor host responses that allow the host to survive and reproduce in the presence of the local parasite, responses that may not be appropriate for parasites from other locations [18].

It is important from the standpoint of transmission to understand the factors dictating the duration of time that an infected snail can survive and persist in producing cercariae. Of particular interest with respect to survivorship of wild snails exposed to wild *S. mansoni* is how heterogeneous the survival rates of the exposed snails are. For example, do many of the exposed snails die before they reach patency and thus resemble female mosquitoes that often perish before they can transmit malaria? Do some snails show an extraordinary ability to withstand the effects of infection and survive considerably longer than other infected snails? Such snails could potentially have a disproportionate effect on maintaining transmission, especially in areas where the human population is being regularly treated for schistosome infection, treatments which, by diminishing egg input into the environment, would also have the effect of reducing the numbers of new snail infections being initiated.

In this study, we investigated the compatibility of *S. mansoni* and its intermediate host *B. pfeifferi*. Both parasites and snails were taken straight from natural habitats from two different localities in Kenya, separated by 300 km. All snails were subsequently maintained in western Kenya in a screened, shaded enclosure subject to ambient temperature and light conditions.

## Methods

### Experimental design

A reciprocal cross infection experiment was conducted whereby snails from Mwea and Asao were exposed to miracidia from either Mwea or Asao (2 homologous or sympatric and 2 heterologous or allopatric combinations). For each combination, 50 screened and trematode-negative 6-9 mm snails were exposed to infection. This was repeated for doses of 1, 5, 10 or 25 miracidia. Two groups each of 50 snails, one from Asao and one from Mwea were not exposed to the parasite and served as negative controls. In total, 900 snails and 8200 miracidia were used.

Observations were made once a week over a period of at least 25 weeks. Snails were counted, screened for schistosome infection by the “shedding” method, and the number of surviving snails recorded. All snails in groups that had shedders surviving beyond 25 weeks of the experiment were observed weekly until they died.

### Parasite and snail sources

*S. mansoni* eggs were obtained from pooled faecal samples from 5 school children aged 6–12 years from both Mukuo primary school in Mwea, Kirinyanga County, central Kenya (00° 40′ 54″S, 037° 20′ 36″E, altitude 1098 m), and Obuon primary school in Asao, Nyakach area, Kisumu County, western Kenya (00° 19′ 01″S, 035° 00′ 22″E, altitude 1171 m). In Asao, *B. pfeifferi* were collected from Asao stream approximately 600 m from Obuon primary school. In Mwea, the snails were collected from Mukou stream, approximately 100 m from Mukou primary school.

### Snail collection and maintenance

Snails were collected at random from a single continuous stretch of each stream using scoops made from a stainless steel sieve with a mesh size of 2 × 2 mm, supported on an iron frame and mounted on a 1.5 m long wooden handle. Snails were sorted out into species based on shell characteristics, using standard taxonomic identification keys [4,19]. Snails identified as *B. pfeifferi* were placed individually in wells of 24-well plastic culture plates containing 1 ml de-chlorinated water, and left on the bench for 2 hrs in indirect sunlight to induce shedding of cercariae. Snails measuring 6-9 mm in shell diameter that did not shed any cercariae were transferred into aquaria (50 snails per aquarium), and allowed to adapt for 5 days before the experiment was started. Prior to exposure of the snails, and subsequently, on a weekly basis during the infection pre-patent period, all the snails were again screened for field-acquired trematode infections. Any snails that shed cercariae during this period were excluded from the experiment and further analysis.

The snails were held in plastic aquaria measuring 45 cm long × 30 cm wide × 15 cm deep in out-door, “semi-field” ambient conditions in a roofed, open-sided, screened structure on KEMRI grounds at the Center for Global Health Research (CGHR), Kisian, Kisumu. Sterile oyster shell crushed to an approximate size of 1 cm × 1 cm covered the bottom of each aquarium. Plants and green algae collected from Asao stream were also introduced into each aquarium, each filled half-way with Asao stream water mixed with dechlorinated tap water (1:1 ratio). Aeration was provided by plastic tubing connected to an air pump. The snails were fed on lightly boiled lettuce supplemented with fish meal as a source of calcium and protein. The goal was to promote more natural survivorship rates of snails by using environmental conditions which were much closer to what snails experience in the field. Water was changed in the aquaria once every week. Temperature ranged from a minimum average of 17°C at night to a maximum average of 31°C during the day.

### Faecal sample collection and recovery of *S. mansoni* eggs and miracidia

Eggs from each locality were isolated by homogenizing the pooled fecal sample using dechlorinated water, followed by filtering through a series of nested sieves of different pore sizes in descending order (710 μM, 425 μM, 212 μM, and 45 μM). Eggs retained by the 45 μM sieve were washed off and placed in a glass conical flask that contained dechlorinated tap water. Flasks were placed under indirect sunlight to allow hatching. For ease of collecting miracidia, each flask was covered with black polythene paper to the neck level to allow collection of the positively phototrophic miracidia in the flask’s neck. Miracidia were transferred to a glass Petri dish and, with the aid of a dissecting microscope, were collected using a pipette for exposure to snails.

### Ethical approval

Approval was obtained from KEMRI Ethics Review Committee (ERC) and the UNM Institutional Review Board (IRB) for all aspects of this project involving human subjects. Children were selected for the study because they are the most vulnerable to schistosomiasis, contribute significantly to environment contamination and parasite transmission, and are easily accessible from their schools. Prior to recruitment, the study team met with the village administration, schools administration, and parents to explain the purpose of the study. The study was explained in a language understandable by the local residents. Participation was voluntary and participants were allowed to withdraw at any time, without penalty. Written and signed consent was sought from parents/guardians, and assent from children above 12 years of age. Involvement of human subjects in this project was limited to provision of faecal samples. Any child found positive for *S. mansoni* was offered standard treatment with praziquantel (40 mg/kg body weight). Children found positive for geohelminths (*Ascaris*, hookworm and *Trichuris*) were offered treatment with albendazole (500 mg) by a trained and qualified clinician. If other medical conditions were detected or suspected, the participant was referred to the nearest hospital for further medical care. To ensure confidentiality, each participant was given a personal identification number as an identifier, and all references to information/data obtained from the participant was referred to by this number. Consent forms, information and data obtained from the study participants were stored securely within KEMRI on password-protected computers.

### Statistical analyses

Data analysis was conducted using IBM SPSS version 21.0 statistical software. Descriptive statistics such as proportions were used to summarize categorical variables while measures of central tendency such as mean, standard error, and range were used to summarize continuous variables. Pearson’s Chi-square test and Fisher exact test were used to test for the association between the dependent variables (mortality and infection) and independent variables (snail source, parasite source and miracidia dose). Odds Ratio (OR) and 95% Confidence Interval (CI) were used to estimate the strength of association between independent variables and each of the dependent variables. Survival rates were examined by Kaplan–Meier survival analysis and survival curves were compared using the log rank test. Cox Proportional Hazards regression was used to determine factors associated with mortality of snails during the 25 weeks of follow-up. A p-value <0.05 was considered statistically significant.

## Results

### Infection rates 4 and 5 weeks post exposure

Because we used field snails with unknown history, we checked for the presence of pre-existing infections and any snail that shed cercariae before the pre-patent period was over was excluded from the study. As expected, none of the snails in the unexposed negative control groups became infected. At 4 weeks post exposure (PE) to *S. mansoni*, sympatric combinations from Asao (Asao snails-Asao parasite) had higher infection rates of 5.1% - 11.7% than the combination of Asao snails and Mwea parasites, which had infection rates of 2.8% - 6.7%. Most Mwea snails had not started shedding cercariae by 4 weeks PE; only sympatric combinations (Mwea snails–Mwea parasite) exposed to 1 or 5 miracidia had shed, with infection rates of 2.9% and 18.6%, respectively.

**Figure 1:**
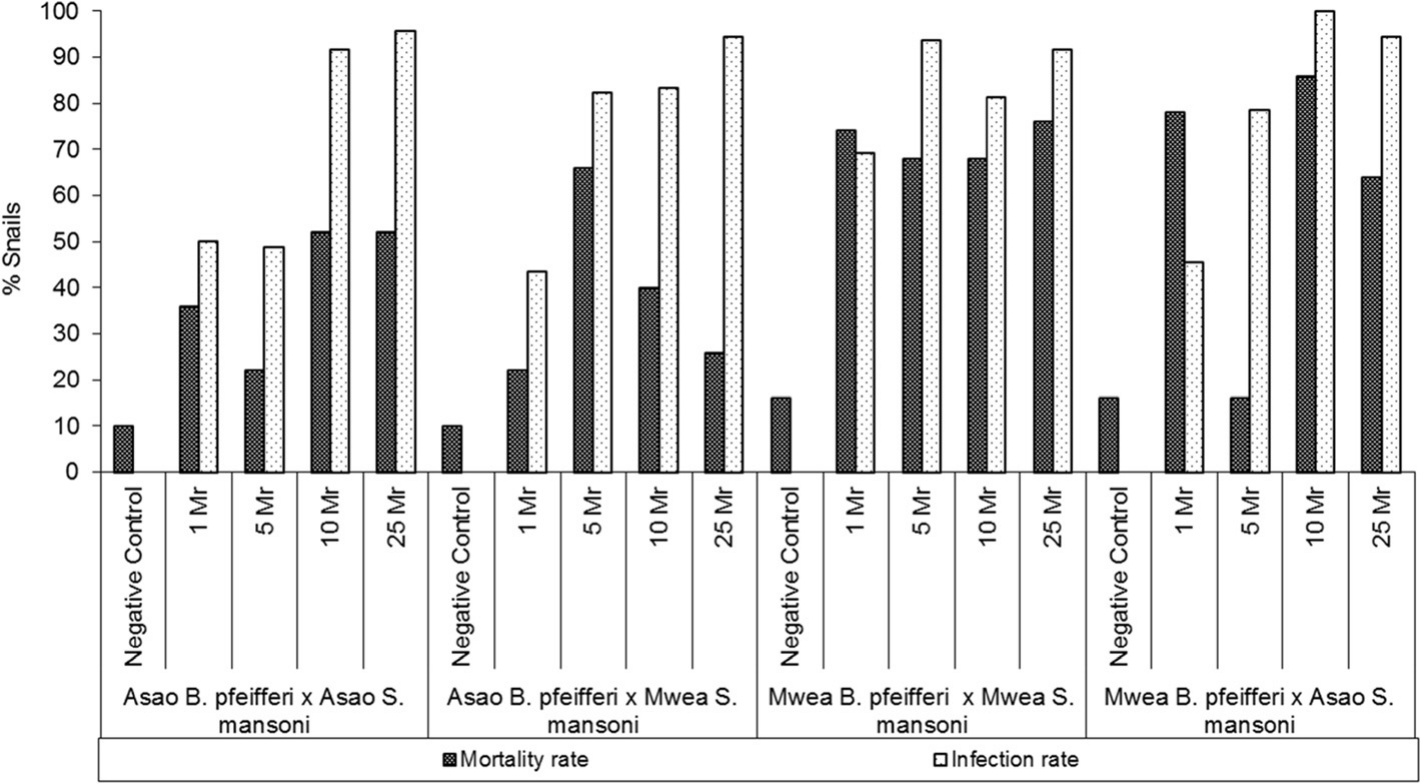
**Mortality and infection rates of the four parasite-snail combinations exposed to different miracidia dose at 5 weeks post exposure (PE).** Mr = miracidia, Infection rate = Number of infected snails/Number of surviving snails at 5 weeks PE.

Snails from all 16 snail-parasite combinations attained their peak infection rates at 5 weeks PE (Figure 1); no newly shedding snails were observed after 5 weeks PE. After 5 weeks there was a gradual but steady decrease of shedders throughout the follow-up period. This decline was due exclusively to mortality of infected snails, and not to self-cures (cessation in shedding). Out of 464 snails that were still alive at 5 weeks PE, overall infection stood at 61.0% (see Additional file 1: Table S1). Generally, infection rates increased with increase in dose of miracidia in the different combinations. Overall, relative to the one miracidium dose infection rate (49.5%), the proportion of snails infected at higher doses was significantly higher: 5 miracidia (71.1%, OR = 2.51 [95% CI = 1.42 – 4.44]; p = 0.002); 10 miracidia (87.0%, OR = 6.84 [95% CI = 3.15 – 14.88]; p < 0.001) and 25 miracidia (96.7%, OR = 29.96 [95% CI = 8.85 – 101.37]; p < 0.001).

### Snail mortality 5 and 10 weeks post exposure

Figure 1 shows mortality and infection rates of snails from the two locations at 5 weeks post exposure (PE), each exposed to allopatric or sympatric *S. mansoni* (see also Additional file 2: Table S2). In general, for those snails exposed to *S. mansoni*, mortality was conspicuously higher for snails from Mwea than for snails collected from Asao. It is noteworthy that survival of the unexposed negative control snails from the 2 localities was not significantly different (84% for Mwea snails versus 90% for Asao snails) at 5 weeks PE, and 74% for Mwea and Asao snails at 10 weeks PE. This suggests that general climatic conditions or physico-chemical properties of the water experienced by the Mwea snails in Kisian were not responsible for their reduced survival following exposure to *S. mansoni*.

Overall mortality by 5 weeks PE was 48.4%. Including all snails in an initial analysis, overall snail mortality as a function of infection revealed a significant association. Mortality among the negative control snails from both Asao and Mwea was significantly lower (13.0%) compared to snails exposed to 1 miracidium (52.5%), (Adjusted OR = 0.12 [95% CI = 0.06 – 0.23]; p < 0.001). A snail not exposed to any miracidia (negative control) was 88.0% less likely to die compared to one exposed to 1 miracidium. Relative to snails exposed to one miracidium, there was no significant increase in mortality when all snails exposed to 5, 10, and 25 miracidia were considered together. However, relative to snails exposed to one miracidium, the risk of mortality increased for snails exposed to 10 miracidia (AOR = 1.45 [95% CI = 0.97 – 2.15]; p = 0.069) or to 25 miracidia (AOR = 1.08 [95% CI = 0.73 – 1.61]; p = 0.688).

**Figure 2:**
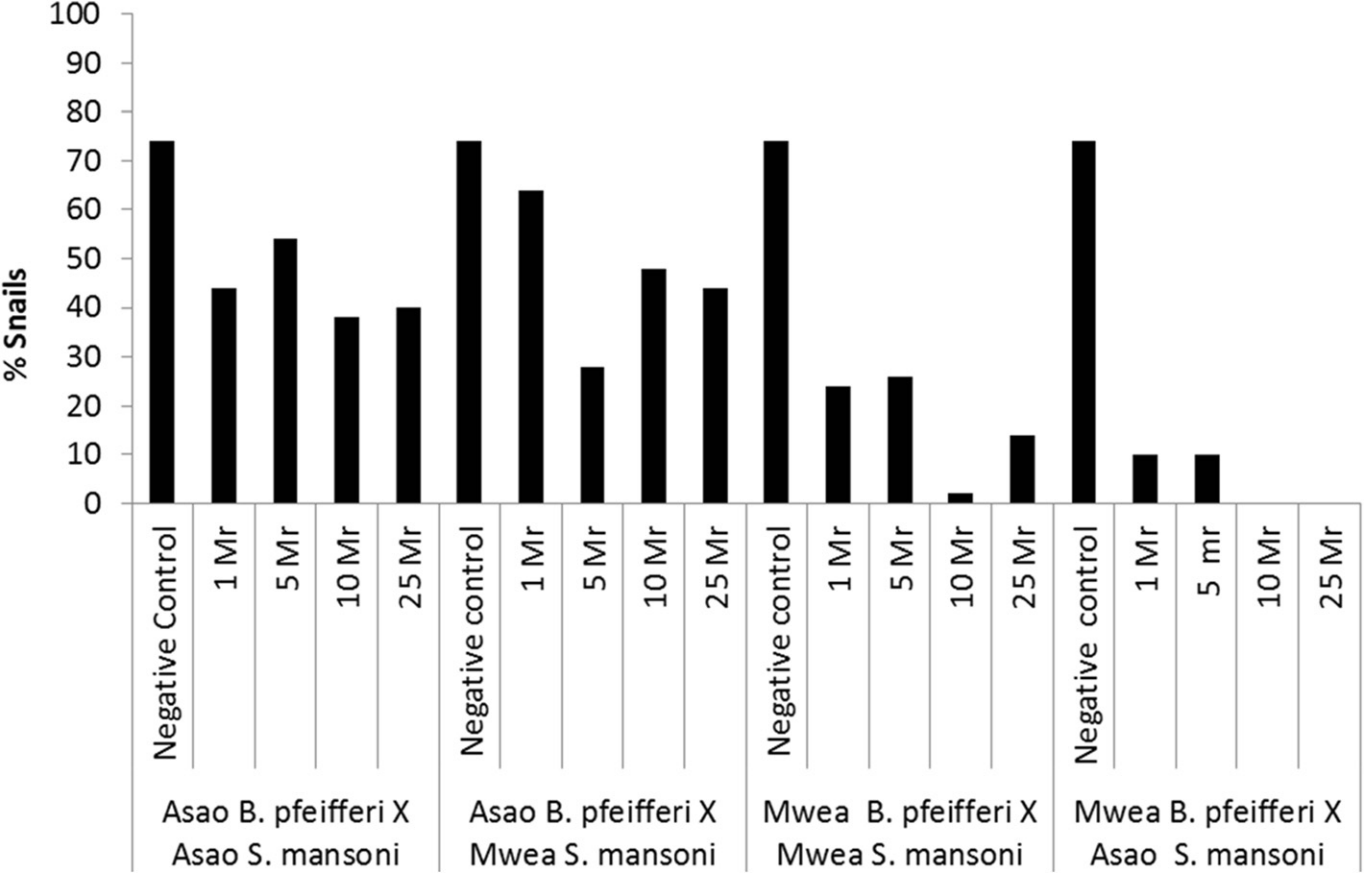
**Survival rate of the different snail–parasite combinations at 10 weeks post exposure to different dose of miracidia.**

To provide a view of the effect of infection on the snails early during the patent period, mortality at 10 weeks PE was also analyzed (see Additional file 3: Table S3 and Figure 2). Overall mortality of all snails at 10 weeks PE was 67%. Mortality among the negative control snails from both Asao and Mwea was significantly lower (26.0%), (OR = 0.11 [95% CI = 0.11 – 0.34]; p < 0.001) compared to snails exposed to 1 miracidium (64.5%), or to higher doses. A snail not exposed to any miracidia (negative control) was 81.0% less likely to die than one exposed to 1 miracidium. Relative to snails exposed to one miracidium, there was no significant increase in mortality for the snails exposed to 5 miracidia, regardless of the snail or miracidia source (70.5%), (OR = 1.32 [95% CI = 0.85 – 0.2.05]; p < 0.200). However, there was a significant increase in mortality for the snails exposed to 10 miracidia (78.0%), (OR = 1.95 [95% CI = 1.22 – 3.11]; p < 0.003) or those exposed to 25 miracidia (75.0%), (OR = 1.70 [95% CI = 1.08 – 2.68]; p < 0.016).

### Snail survival 25 weeks following exposure to miracidia

**Figure 3:**
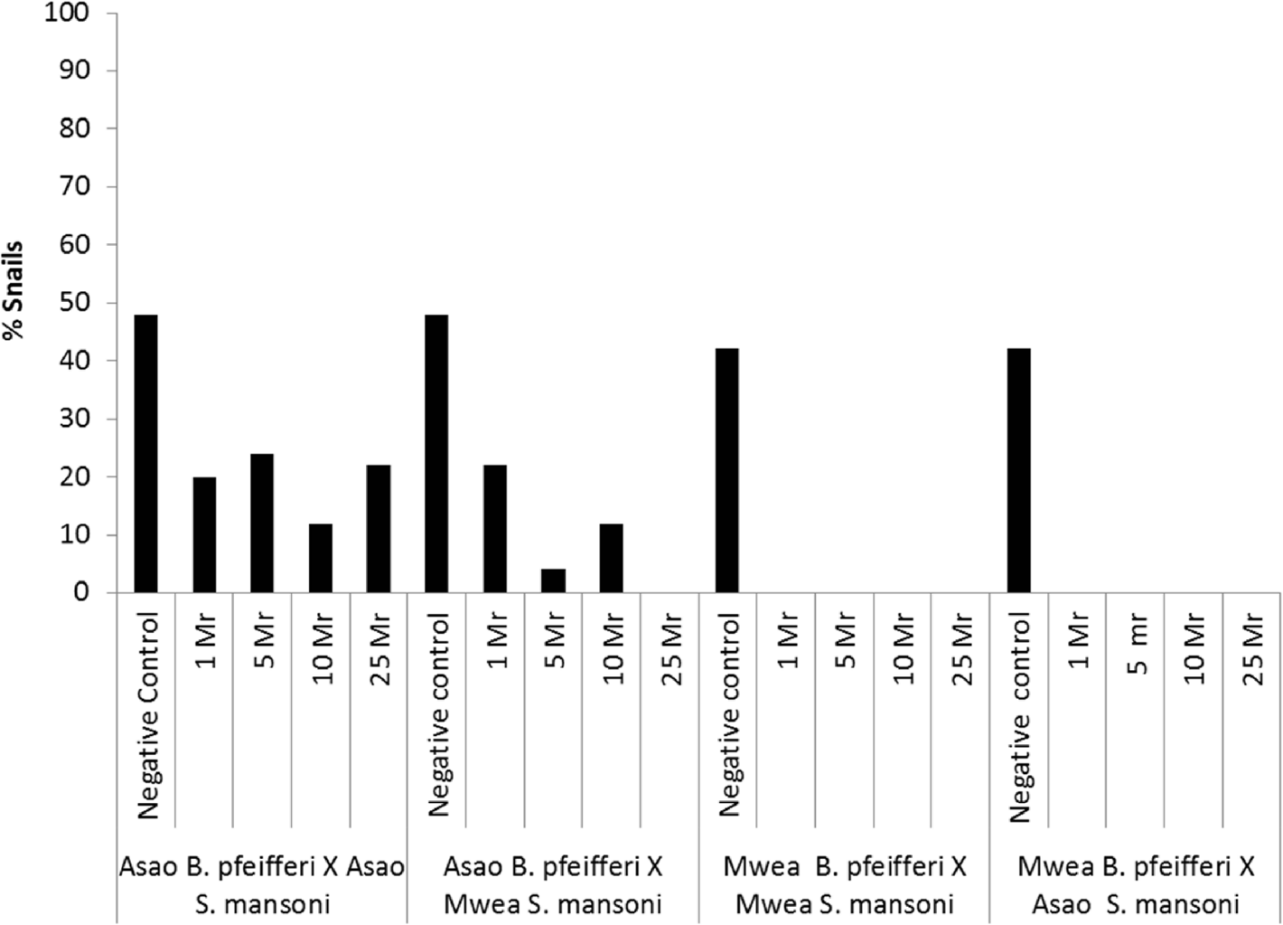
**Survival rate of the different snail–parasite combinations for the 25 weeks of follow-up post exposure to different dose of miracidia.**

Survival of snails for the 25 weeks of follow-up was highest among the two negative control groups from Asao and Mwea (48% and 40% respectively). Asao snails exposed to low miracidia doses (1 or 5) had a relatively higher survival rate (>20%) compared to Mwea snails which had all died by the end of follow up period (Figure 3).

Table 1 presents mean survival times for Mwea and Asao snails exposed to homologous or heterologous *S. mansoni*. Overall mean survival time was higher in Asao snails, with those exposed to homologous *S. mansoni* having mean survival of 12.06 [95% CI = 10.68 – 13.44] weeks and those exposed to heterologous *S. mansoni* having 12.68 [95% CI = 11.63 – 13.73] weeks. For Mwea snails, survival period were 5.51 [95% CI = 4.83 – 6.81] weeks and 4.70 [95% CI = 4.28 – 5.11] weeks for those exposed to homologous and heterologous *S. mansoni*, respectively. When viewing all snail-parasite combinations, mean survival time decreased progressively with increase in miracidia dose. Overall there was no marked difference in mean survival times in relation to source of parasite from the two locations (Mwea or Asao).

**Table 1:** **Mean survival time for snails exposed to different homologous or heterologous doses of** ***S. mansoni*** **during 25 weeks of follow-up**

**Miracidia** ^**1**^	**Mean** ^**2**^	**Std. Error**	**95% CI**	
			**Lower**	**Upper**
**Mwea** ***B. pfeifferi*** **x Mwea** ***S. mansoni***				
1 miracidium	7.66	0.80	6.09	9.23
5 miracidia	6.18	0.80	4.61	7.75
10 miracidia	4.04	0.43	3.20	4.88
25 miracidia	4.14	0.55	3.05	5.23
**Overall**	**5.51**	**0.35**	**4.83**	**6.18**
**Mwea** ***B. pfeifferi*** **x Asao** ***S. mansoni***				
1 miracidium	5.10	0.58	3.96	6.24
5 miracidia	5.34	0.38	4.59	6.09
10 miracidia	3.94	0.26	3.43	4.45
25 miracidia	4.40	0.37	3.68	5.12
**Overall**	**4.70**	**0.21**	**4.28**	**5.11**
**Asao** ***B. pfeifferi*** **x Mwea** ***S. mansoni***				
1 miracidium	14.42	1.34	11.80	17.04
5 miracidia	13.52	0.88	11.80	15.24
10 miracidia	12.44	1.18	10.13	14.75
25 miracidia	10.30	0.69	8.95	11.65
**Overall**	**12.68**	**0.54**	**11.63**	**13.73**
**Asao** ***B. pfeifferi*** **x Asao** ***S. mansoni***				
1 miracidium	12.70	1.35	10.05	15.35
5 miracidia	14.18	1.26	11.71	16.65
10 miracidia	9.62	1.25	7.17	12.07
25 miracidia	10.98	1.49	8.06	13.90
**Overall**	**12.06**	**0.70**	**10.68**	**13.44**
**Overall analysis**				
Negative control	18.34	0.86	16.65	20.03
1 miracidium	9.97	0.60	8.80	11.14
5 miracidium	9.81	0.53	8.78	10.84
10 miracidia	7.51	0.52	6.50	8.52
25 miracidia	7.46	0.50	6.48	8.43

^1^No. of snails per miracidia dose was 50; ^2^Mean snail survival (in weeks).

**Figure 4:**
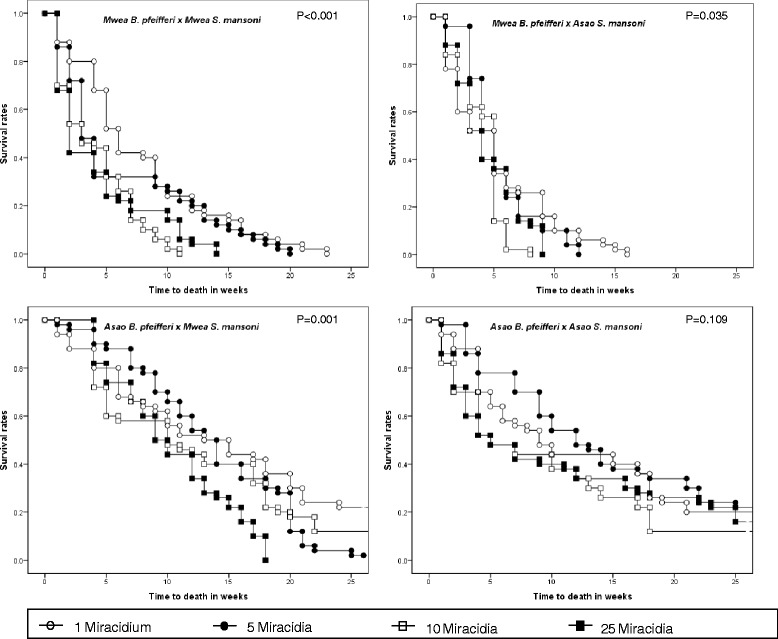
**Kaplan-Meier survival curves for snails (** ***B. pfeifferi*** **) from Asao and Mwea exposed to different doses of homologous or heterologous** ***S. mansoni*** **miracidia, during 25 weeks of follow-up PE.**

Comparison of survival curves by log rank test for each snail-parasite combination and the 4 different miracidia doses is shown in Figure 4. Except for the Asao *B. pfeifferi* x Asao *S. mansoni* combination, all other combinations showed significant differences (p < 0.05) in survival curves for snails exposed to the 4 different miracidia doses.

### Factors associated with mortality among the snails during 25 weeks of follow-up

Cox proportional hazards regression was used to analyze the risk factors for death among the snails (Table 2). Adjusting for the source of snail, the dose of miracidia was significantly associated with mortality of snails. Compared to the group exposed to one miracidium, unexposed controls were significantly less likely to die (Adjusted HR = 0.26 [95% CI = 0.19 – 0.36]; p < 0.001). A snail not exposed to miracidia was 74% less likely to die compared to a snail exposed to one miracidium. Relative to the group exposed to one miracidium, increased risk of mortality was significantly associated with exposure to 10 miracidia (Adjusted HR = 1.52 [95% CI = 1.23 – 1.87]; p < 0.001) and 25 miracidia (Adjusted HR = 1.55 [95% CI = 1.26 – 1.80]; p < 0.001). A snail exposed to 10 miracidia or 25 miracidia was 1.52 times or 1.55 times, respectively, more likely to die compared to one exposed to one miracidium. Overall, Mwea as the source of snails was significantly associated with increased incidence of death compared to Asao (Adjusted HR = 2.86 [95% CI = 2.45 – 3.34]; p < 0.001).

**Table 2:** **Analysis of factors associated with mortality of snails after 25 weeks of follow-up**

**Variables**	**Bivariate analysis**				**Multivariate analysis**			
	**HR** ^**¥**^	**95% CI** ^**€**^		**P value**	**Adjusted HR**	**95% CI**		**P value**
		**Lower**	**Upper**			**Lower**	**Upper**	
**Miracidia**								
Negative control	0.35	0.26	0.47	**<0.001**	0.26	0.19	0.36	**<0.001**
Miracidia 1	1.00				1.00			
Miracidia 5	1.05	0.85	1.29	0.648	1.11	0.90	1.36	0.336
Miracidia 10	1.36	1.11	1.67	**0.003**	1.52	1.23	1.87	**<0.001**
Miracidia 25	1.41	1.15	1.73	**0.001**	1.55	1.26	1.90	**<0.001**
**Snail source**								
Mwea BP	2.19	1.90	2.53	**<0.001**	2.86	2.45	3.34	**<0.001**
Asao BP	1.00				1.00			
**Parasite source**								
Mwea SM	0.98	0.85	1.12	0.737				
Asao SM	1.00							

^**¥**^Hazard Ratio; ^€^95% Confidence Interval.

**Figure 5:**
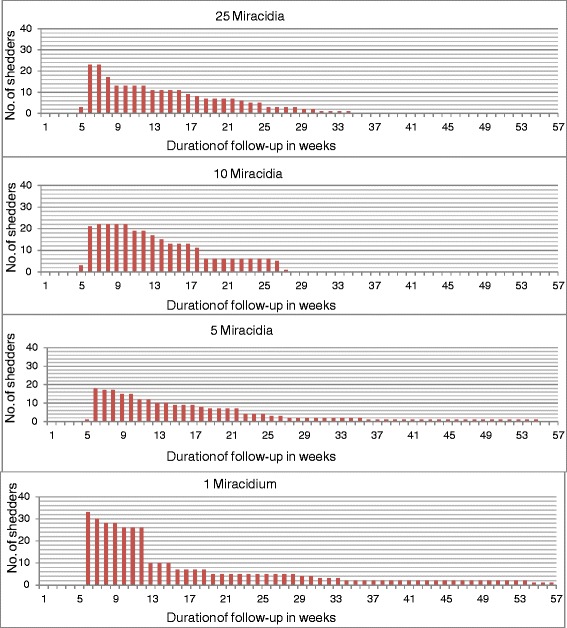
**Number of shedders for Asao** ***B. pfeifferi*** **exposed to different numbers of Asao** ***S. mansoni*** **miracidia in 58 weeks follow-up.**

At the end of the 25 weeks period of observation, a significant proportion of the infected snails in some treatment combinations were still alive. For Asao snails, except for the heterologous group exposed to 25 miracidia which had all died, the remaining seven groups had between 4% - 24% survivors. Given the epidemiological significance of longevity of infected snails, observation was continued to determine how long these snails would survive in our outdoor aquaria. For the Asao homologous combination, Figure 5 presents the number of shedders as a function of time since first exposure. Note that for all dosage groups, some infected snails survived for at least half a year. Among those exposed to 25 miracidia, 1 out of 50 survived up to week 35, and of those exposed to 10 miracidia, 1 out of 50 survived up to week 28. Remarkably, for the lower dose groups, 2 out of 50 snails exposed to 1 miracidia, and 1 out of 50 snails exposed to 5 miracidia survived for 58 weeks, shedding cercariae throughout the patent period. These snails also grew to the relatively large size of 14 mm shell diameter. All snails were 6-9 mm in shell diameter when exposed so were already young adults at the time.

## Discussion

Compared to other studies assessing the interactions between *B. pfeifferi* and *S. mansoni* [18,20-26] this study is distinctive in 1) focusing on snails and parasites of Kenyan origin; 2) using snails taken straight from the field rather than lab-reared snails for the experiments described; 3) using *S. mansoni* miracidia derived from pooled samples from infected school children rather than miracidia derived from lab-adapted strains of *S. mansoni* maintained in rodents; and 4) maintaining all snails in aquaria which were held in a roofed shelter with open, screened sides that permitted exposure of aquaria to natural light fluctuations and ambient temperatures. Our overall goal was to provide study conditions as close as possible to conditions occurring in nearby natural habitats, even if it meant sacrificing control over some variables such as temperature. One of the costs of pursuing this approach is that the snails collected from nature had only 5 days to adapt to our rearing conditions before they were used in experiments. Consequently, subsequent mortality in some snail groups was higher than might be expected had the snails we used been the progeny of lab-adapted snails. Another cost is that the exact history of trematode exposure (including schistosomes) of the snails we used, prior to exposing them to schistosome miracidia in the present experiment, is unknown. However, the presence of pre-existing trematode infections in our snails was determined both by screening the snails right after collection, and subsequently, by screening them on a weekly basis, to detect infections not yet patent at the time we set up the experiment. Through this process, only 0.5% shed field-acquired trematode cercariae, and these were excluded from the experiment. Consequently, these did not complicate our experiments significantly. We note though that our use of field-derived snails and schistosomes has the advantage of minimizing the genetic bottlenecking effects that occur quickly once both snails and schistosomes are brought into domestication [27,28].

Our study used a reciprocal cross infection design involving *B. pfeifferi* and *S. mansoni* derived from both Asao and Mwea. Such a design has been advocated for optimal detection of local adaptation effects [12]. This design, coupled with the use of field-derived snails, offers some logistical challenges. In our case, snails collected at Mwea had to be brought to our snail-rearing facilities in Kisian, western Kenya, a distance of approximately 300 km. Stresses involved in transport, exposure to a different climatic regime and aquarium water in Kisian, and isolation and exposure to infection are likely to have contributed to the observed overall higher mortality of *S. mansoni*-exposed snails from Mwea. In spite of these problems, we nonetheless feel it is important to undertake at least some compatibility experiments that mimic nature more closely, in which schistosomes confront snails that in many respects are complex holobionts, with gut floras and complements of both external and internal symbionts, that are bound to differ from those present in snails that have been reared in the lab for one or more generations. Also, although the exposure of snails to the variable conditions experienced in our outdoor aquaria might be questioned as something that would merely create noise in our experimental system, once field-derived snails adjusted to the outdoor aquaria, their survival was in some cases remarkably long. We consequently feel our approach is justified and worthy of consideration as an alternative to the standard lab-based and more controlled approach to study snail-schistosome interactions, one that provides a much closer approximation to the realities of natural schistosome transmission foci. A further refinement would be to use water and plants in rearing aquaria derived from the source area for snails (as we did for snails from Asao) though this poses obvious logistical problems when the snails are from distant locations, such as for the snails from Mwea.

Using the exposure of snails to one miracidium per snail as a sensitive and widely used measure of compatibility, we note that the infection rates we observed in our snails ranged from 43 to 69%, with the two heterologous combinations of snails and parasites yielding slightly lower infection rates than either homologous combinations. Other studies using this host parasite system and similar exposure doses have reported lower (32%) values [21], similar (46.4-56.3%) values [24,18] or higher (80.8-87%) values [23,18]. The highest value reported is of Senegalese *B. pfeifferi* exposed to Senegalese *S. mansoni*, a unique situation in which a major habitat change may have favored rapid expansion of a genetically uniform and susceptible population of *B. pfeifferi* [29].

As is similar with most other studies of exposure of snails to increasing numbers of schistosome miracidia, infection rates were observed to increase with dose, achieving 100% in one combination. Usually, doses of 5 miracidia per snail or higher produced infection rates of 75-95%, with no obvious tendency for homologous combinations to yield higher infection rates than heterologous combinations. Mortality also increased with miracidia dose. Mean longevity was significantly longer for snails exposed to 1 miracidium (9.97 weeks) followed by those exposed to 5 miracidia (9.81 weeks), with the lowest mean longevity being for snails exposed to 25 miracidia (7.46 weeks). Mwea and Asao parasites did not differ obviously in their likelihood of causing snail death. Increasing mortality with higher doses of miracidia is also a commonly observed trend in studies of experimental exposure of snails to schistosome infection, including the *B. pfeifferi-S. mansoni* combination [21-24].

Our study of local adaptation was complicated by the higher mortality rates exhibited by snails from Mwea, but provided no overall statistical evidence to suggest that sympatric combinations of snails and schistosomes yielded either higher or lower infection rates than allopatric combinations. Mwea snails and parasites had higher infection rates at 1 and 5 miracidia/snail doses than other combinations, and Asao snails and parasites had higher infection rates at 10 and 25 miracidia/snail doses, but the differences were not dramatic and heterologous combinations also yielded high infection rates. There was some suggestion that sympatric combinations had shorter prepatent periods than allopatric combinations but the effect was not strong. As noted, the sympatric Asao combination produced a few infected snails capable of long survival times, suggestive of local adaptation of the parasite to the snail. With respect to snail mortality, there was no convincing evidence that mortality rates were either consistently lower or higher for sympatric than for allopatric combinations of snails and parasites. In our system, as assessed primarily by infection rates and snail mortality rates, local adaptation was not a prominent feature for either schistosomes or snails.

Studies of local adaptation between *Biomphalaria* snails and *S. mansoni* have yielded mixed results [12]. In an extensive study of 8 *Biomphalaria* species and 34 different populations, exposed to 7 different isolates of *S. mansoni*, Frandsen [26] concluded that local combinations of hosts and parasites were often not the most compatible. This was particularly true for species such as *B. glabrata* or *B. pfeifferi* known for their broad susceptibility to *S. mansoni* from a variety of origins. For other species of *Biomphalaria*, such as *B. alexandrina* from Egypt or *B. glabrata* from the West Indies, compatibility with local isolates of *S. mansoni* was more pronounced. A more recent study [14] of local adaptation involving *B. glabrata* populations from the island of Guadeloupe and *S. mansoni* circulating in each snail population found no evidence for local adaptation in either snail or parasite. This study was similar to ours in that it used both snails and schistosomes derived from the field (the eggs came from naturally infected *Rattus rattus*). For this study, the distances between snail populations were much less than for the two populations we studied. The authors noted that the populations of snails and schistosomes they studied were subject to frequent drastic fluctuations and this may have prevented coevolutionary associations from developing. They also found migration rates of snails and parasites in their system to be similar which may also have prevented local adaptations from developing. Another more recent study in West Africa [18] using *S. mansoni* from Benin and 3 lab populations of *B. pfeifferi* from Benin and two from Oman (over 5,000 km from Benin) did reveal local adaptation. However, most of the combinations tested including one of the Oman populations exhibited over 80% infection rates following exposure to one miracidium per snail. These authors used a more complex measure of local adaptation, taking into account length of pre-patent period, rates of cercarial production, snail mortality, and snail fecundity during the pre-patent period.

The lack of obvious local adaptation in our study may be for some of the same reasons invoked by the study in Guadeloupe [14] which concluded that genetic drift, extinction-recolonization processes, host and parasite dispersal abilities and selection in different directions or intensity all affect co-evolutionary dynamics. Both snail habitats we sampled experience considerable seasonal variation with respect to water volume, including with occasional flooding. As *B. pfeifferi* is a strong preferential self-fertilizer, it seems likely that populations that re-colonize sections of habitats scoured of snails by flooding may represent a different clonal population than pre-flood populations. In other words, it is reasonable to expect that snail populations vary considerably over time in our study sites. With respect to *S. mansoni*, in both locations studied, there have been some efforts directed towards control using praziquantel, and although this control has mostly targeted school children and has not had a major effect on limiting transmission, it may well have an effect on breaking up co-evolutionary associations with local hosts. It is possible that the pool of available *S. mansoni* genotypes will be altered by control programs. Movements of infected people transporting S*. mansoni* into and out of local snail populations can be a strong factor disrupting local adaptation between parasite and snail [30,31]. It is not likely human-facilitated movement of *S. mansoni* was a significant factor in this study involving two small rural villages separated by 300 km. There are no distinctive economic or social ties of which we are aware that would favor movement between the two villages.

As a final thought on the topic of local adaptation, several studies have shown *B. pfeifferi* to be generally susceptible to *S. mansoni* regardless of the geographic origin of the latter [26]. For example, we recently noted that *B. pfeifferi* removed from the field from Kenya for only a couple of generations were readily infected with the PR1 strain of *S. mansoni* (originating from Puerto Rico) that has been maintained in the laboratory for at least 50 years in rodents and *B. glabrata*. Our Kenyan snails have never encountered a strain of *S. mansoni* originating from the West Indies yet readily succumbed to infection. Given this inherent susceptibility, it seems any local adaptation that might occur, especially given the difficulties of preserving the necessary co-evolutionary associations, particularly when the snails involved also have to cope with other parasite species including other equally common trematode species, may not have much explanatory power in terms of understanding schistosome transmission dynamics.

Lastly, it is noteworthy that in the Asao-Asao combination of snails and schistosomes we studied, some of the snails exposed to low doses (1 or 5) of miracidia continued to shed cercariae for over a year (58 weeks). One study of the survival of *B. glabrata* exposed to *S. mansoni* has reported survival of infected snails for over a year [32], but laboratory studies examining survival of *B. pfeifferi* report maximum survival times of between 51 and 239 days [18,20,22-26]. The lengthy survival time of over one year is noteworthy because the snails were already young adults when exposed to infection. Our use of outdoor aquaria may have precluded us from tightly controlling important variables like temperature, but we feel they have also provided good conditions in which snails can thrive once adjusted to them, giving us, in our opinion, a more natural view of snail longevity. Estimates of adult life expectancy for *B. pfeifferi* in the field are much shorter than for laboratory-reared snails, with estimates typically ranging from 2 to 7 weeks [7,33]. Although it is certainly true that many factors would conspire to limit survivorship of snails in the field, it is possible that our ability to measure this accurately may be limited. The low survivorships rates may be based on mark-recapture studies [34] which may return low rates of marked snails from complex aquatic habitats. Also, in some cases, we recover infected snails from the field that are unusually large and/or covered with algae and other periphyton that convey the impression of considerable age. So, it does not seem inconceivable to us that some infected snails may persist for many months in natural habitats. As prolonged infection and shedding is a potential measure of local adaptation of parasite to host [18], it is perhaps not surprising that we reported it in one of our homologous combinations.

## Conclusions

Although high miracidial doses ensure high infection rates, such snails tend to die faster and if such infections are achieved in nature, as they sometimes are [35], they may not be the longest-term transmitters of infection. Conversely, snails receiving lower miracidial dose infections survive for longer periods, at least in experimental infections, and could potentially transmit the parasite longer. If some proportion of “super-survivor” snails occur in the field, they could pose problems for schistosomiasis control initiatives which rely mostly on mass drug administration, a strategy that leaves populations of snails and the larval schistosomes they contain unmolested. Even if drug treatment were timed more frequently than currently practiced (say every 4 or 6 months) as a means to minimize new snail infections more efficiently and lower the eventual rate of reinfections in people, super-survivor snails could potentially continue to persist through multiple rounds of treatment and still be there to initiate reinfections. As the proportion of super-surviving snails would probably be low, this effect may prove to be trivial, but along with contribution of schistosomes eggs by reservoir hosts [36], may favor persistence of schistosomes even in the face of more frequent treatments. It is our view that prospective control programs should incorporate a component to control snails and associated production of cercariae to help sustain the gains made through chemotherapy.

## Additional files

Additional file 1: Table S1. Analysis of snail infection 5 weeks post exposure to miracidia. Additional file 2: Table S2. Analysis of snail mortality 5 weeks post exposure. Additional file 3: Table S3. Analysis of snail mortality 10 weeks post exposure to miracidia.

## Abbreviations

AOR: Adjusted odds ratio
CGHR: Center for global health research
CI: Confidence interval
DBL: Danish Bilharziasis Laboratory
ERC: Ethical Review Committee
HR: Hazards ratio
IRB: Institutional Review Board
KEMRI: Kenya Medical Research Institute
Kg: Kilogram
Kms: Kilometes
Mr: Miracidia
Mg: Milligram
OR: Odds ratio
PE: Post exposure
UD: Undefined
UNM: University of New Mexico
WHO: World health organization
μM: Micrometer
