# Supplementary material for: Field-derived Schistosoma mansoni and Biomphalaria pfeifferi in Kenya: a compatible association characterized by lack of strong local adaptation, and presence of some snails able to persistently produce cercariae for over a year
Source: Parasit Vectors. 2014 Nov 26;7:533. doi: 10.1186/s13071-014-0533-3 (PMC4253995; doi:10.1186/s13071-014-0533-3)
Supplement: Additional file 1: Table S1. — Analysis of snail infection 5 weeks post exposure to miracidia. [file 13071_2014_533_MOESM1_ESM.docx]

Table S1: Analysis of snail infection 5 weeks post exposure to miracidia

| **Miracidia** | **Infected** | | **Not infected** | | **OR^¥^** | **95% CI^€^** | | **p value** |
| --- | --- | --- | --- | --- | --- | --- | --- | --- |
|  | **n** | **%** | **n** | **%** |  | **Lower** | **Upper** |  |
| **Mwea *B. pfeifferi* x Mwea *S. mansoni*** | |  |  |  |  |  |  |  |
| Negative control | 0 | 0.0% | 42 | 100.0% | UD | UD | UD | 0.997 |
| 1 miracidium | 9 | 69.2% | 4 | 30.8% | 1.00 |  |  |  |
| 5 miracidia | 15 | 93.8% | 1 | 6.3% | 6.67 | 0.64 | 69.34 | 0.112 |
| 10 miracidia | 13 | 81.3% | 3 | 18.8% | 1.93 | 0.34 | 10.77 | 0.456 |
| 25 miracidia | 11 | 91.7% | 1 | 8.3% | 4.89 | 0.46 | 51.87 | 0.188 |
| Total | 48 | 48.5% | 51 | 51.5% |  |  |  |  |
| **Mwea *B. pfeifferi* x Asao *S. mansoni*** | |  |  |  |  |  |  |  |
| 1 miracidium | 5 | 45.5% | 6 | 54.5% | 1.00 |  |  |  |
| 5 miracidia | 33 | 78.6% | 9 | 21.4% | 4.40 | 1.09 | 17.79 | **0.038** |
| 10 miracidia | 7 | 100.0% | 0 | 0.0% | UD | UD | UD | 0.999 |
| 25 miracidia | 17 | 94.4% | 1 | 5.6% | 20.40 | 1.96 | 211.79 | **0.012** |
| Total | 62 | 79.5% | 16 | 20.5% |  |  |  |  |
| **Asao *B. pfeifferi* x Mwea *S. mansoni*** | |  |  |  |  |  |  |  |
| 1 miracidium | 17 | 43.6% | 22 | 56.4% | 1.00 |  |  |  |
| 5 miracidia | 14 | 82.4% | 3 | 17.6% | 6.04 | 1.49 | 24.45 | **0.012** |
| 10 miracidia | 25 | 83.3% | 5 | 16.7% | 6.47 | 2.05 | 20.44 | **0.001** |
| 25 miracidia | 35 | 94.6% | 2 | 5.4% | 22.65 | 4.76 | 107.69 | **<0.001** |
| Total | 91 | 74.0% | 32 | 26.0% |  |  |  |  |
| **Asao *B. pfeifferi* x Asao *S. mansoni*** | |  |  |  |  |  |  |  |
| Negative control | 0 | 0.0% | 45 | 100.0% | UD | UD | UD | 0.997 |
| 1 miracidium | 16 | 50.0% | 16 | 50.0% | 1.00 |  |  |  |
| 5 miracidia | 19 | 48.7% | 20 | 51.3% | 0.95 | 0.37 | 2.42 | 0.914 |
| 10 miracidia | 22 | 91.7% | 2 | 8.3% | 11.00 | 2.21 | 54.75 | **0.003** |
| 25 miracidia | 23 | 95.8% | 1 | 4.2% | 23.00 | 2.76 | 191.36 | **0.004** |
| Total | 80 | 48.8% | 84 | 51.2% |  |  |  |  |
| **Overall** |  |  |  |  |  |  |  |  |
| Negative control | 0 | 0.0% | 87 | 100.0% | UD | UD | UD | 0.996 |
| 1 miracidium | 47 | 49.5% | 48 | 50.5% | 1.00 |  |  |  |
| 5 miracidia | 81 | 71.1% | 33 | 28.9% | 2.51 | 1.42 | 4.44 | **0.002** |
| 10 miracidia | 67 | 87.0% | 10 | 13.0% | 6.84 | 3.15 | 14.88 | **<0.001** |
| 25 miracidia | 86 | 94.5% | 5 | 5.5% | 17.57 | 6.54 | 47.15 | **<0.001** |
| Total | 281 | 60.6% | 183 | 39.4% |  |  |  |  |

^¥^ - Odds Ratio, ^€^ - 95% Confidence Interval, UD=Undefined
