# Supplementary material for: Field-derived Schistosoma mansoni and Biomphalaria pfeifferi in Kenya: a compatible association characterized by lack of strong local adaptation, and presence of some snails able to persistently produce cercariae for over a year
Source: Parasit Vectors. 2014 Nov 26;7:533. doi: 10.1186/s13071-014-0533-3 (PMC4253995; doi:10.1186/s13071-014-0533-3)
Supplement: Additional file 2: Table S2. — Analysis of snail mortality 5 weeks post exposure. [file 13071_2014_533_MOESM2_ESM.docx]

Table S2: Analysis of snail mortality 5 weeks post exposure

| **Miracidia** | **Dead** | | **Alive** | | **OR^¥^** | **95% CI^€^** | | **p value** |
| --- | --- | --- | --- | --- | --- | --- | --- | --- |
|  | **n** | **%** | **n** | **%** |  | **Lower** | **Upper** |  |
| **Mwea *B. pfeifferi* x Mwea *S. mansoni*** | |  |  |  |  |  |  |  |
| Negative control | 8 | 16.0% | 42 | 84.0% | 0.07 | 0.02 | 0.18 | **<0.001** |
| Miracidia 1 | 37 | 74.0% | 13 | 26.0% | 1.00 |  |  |  |
| Miracidia 5 | 34 | 68.0% | 16 | 32.0% | 0.75 | 0.31 | 1.78 | 0.509 |
| Miracidia 10 | 34 | 68.0% | 16 | 32.0% | 0.75 | 0.31 | 1.78 | 0.509 |
| Miracidia 25 | 38 | 76.0% | 12 | 24.0% | 1.11 | 0.45 | 2.75 | 0.817 |
| Total | 151 | 60.4% | 99 | 39.6% |  |  |  |  |
| **Mwea *B. pfeifferi* x Asao *S. mansoni*** | |  |  |  |  |  |  |  |
| Miracidia 1 | 39 | 78.0% | 11 | 22.0% | 1.00 |  |  |  |
| Miracidia 5 | 8 | 16.0% | 42 | 84.0% | 0.05 | 0.02 | 0.15 | **<0.001** |
| Miracidia 10 | 43 | 86.0% | 7 | 14.0% | 1.73 | 0.61 | 4.91 | 0.301 |
| Miracidia 25 | 32 | 64.0% | 18 | 36.0% | 0.50 | 0.21 | 1.21 | 0.126 |
| Total | 122 | 61.0% | 78 | 39.0% |  |  |  |  |
| **Asao *B. pfeifferi* x Mwea *S. mansoni*** | |  |  |  |  |  |  |  |
| Miracidia 1 | 11 | 22.0% | 39 | 78.0% | 1.00 |  |  |  |
| Miracidia 5 | 33 | 66.0% | 17 | 34.0% | 6.88 | 2.83 | 16.74 | **<0.001** |
| Miracidia 10 | 20 | 40.0% | 30 | 60.0% | 2.36 | 0.98 | 5.68 | 0.054 |
| Miracidia 25 | 13 | 26.0% | 37 | 74.0% | 1.25 | 0.50 | 3.13 | 0.640 |
| Total | 77 | 38.5% | 123 | 61.5% |  |  |  |  |
| **Asao *B. pfeifferi* x Asao *S. mansoni*** | |  |  |  |  |  |  |  |
| Negative control | 5 | 10.0% | 45 | 90.0% | 0.20 | 0.07 | 0.59 | **0.004** |
| Miracidia 1 | 18 | 36.0% | 32 | 64.0% | 1.00 |  |  |  |
| Miracidia 5 | 11 | 22.0% | 39 | 78.0% | 0.50 | 0.21 | 1.21 | 0.126 |
| Miracidia 10 | 26 | 52.0% | 24 | 48.0% | 1.93 | 0.86 | 4.29 | 0.109 |
| Miracidia 25 | 26 | 52.0% | 24 | 48.0% | 1.93 | 0.86 | 4.29 | 0.109 |
| Total | 86 | 34.4% | 164 | 65.6% |  |  |  |  |
| **Overall** |  |  |  |  |  |  |  |  |
| Negative control | 13 | 13.0% | 87 | 87.0% | 0.14 | 0.07 | 0.26 | **<0.001** |
| Miracidia 1 | 105 | 52.5% | 95 | 47.5% | 1.00 |  |  |  |
| Miracidia 5 | 86 | 43.0% | 114 | 57.0% | 0.68 | 0.46 | 1.01 | 0.058 |
| Miracidia 10 | 123 | 61.5% | 77 | 38.5% | 1.45 | 0.97 | 2.15 | 0.069 |
| Miracidia 25 | 109 | 54.5% | 91 | 45.5% | 1.08 | 0.73 | 1.61 | 0.688 |
| Total | 436 | 48.4% | 464 | 51.6% |  |  |  |  |

^¥^ - Odds Ratio; ^€^ - 95% Confidence Interval
