# Supplementary material for: Field-derived Schistosoma mansoni and Biomphalaria pfeifferi in Kenya: a compatible association characterized by lack of strong local adaptation, and presence of some snails able to persistently produce cercariae for over a year
Source: Parasit Vectors. 2014 Nov 26;7:533. doi: 10.1186/s13071-014-0533-3 (PMC4253995; doi:10.1186/s13071-014-0533-3)
Supplement: Additional file 3: Table S3. — Analysis of snail mortality 10 weeks post exposure to miracidia. [file 13071_2014_533_MOESM3_ESM.docx]

Table S3: Analysis of snail mortality 10 weeks post exposure to miracidia

| **Miracidia** | **Dead** | | **Alive** | | **OR^¥^** | **95% CI^€^** | | **p value** |
| --- | --- | --- | --- | --- | --- | --- | --- | --- |
|  | **n** | **%** | **n** | **%** |  | **Lower** | **Upper** |  |
| **Mwea *B. pfeifferi* x Mwea *S. mansoni*** | | | | |  |  |  |  |
| Negative control | 13 | 26.0 | 37 | 74.0 | 0.11 | 0.04 | 0.30 | **<0.001** |
| 1 Mr | 38 | 76.0 | 12 | 24.0 | 1.00 |  |  |  |
| 5 Mr | 37 | 74.0 | 13 | 26.0 | 0.90 | 0.33 | 2.44 | 0.817 |
| 10 Mr | 49 | 98.0 | 1 | 2.0 | 15.47 | 1.93 | 332.50 | **0.001** |
| 25 Mr | 43 | 86.0 | 7 | 14.0 | 1.94 | 0.63 | 6.15 | 0.202 |
| Total | 180 | 72.0 | 70 | 28.0 |  |  |  |  |
| **Mwea *B. pfeifferi* x Asao *S. mansoni*** | | | | |  |  |  |  |
| 1 Mr | 45 | 90.0 | 5 | 10.0 | 1.00 |  |  |  |
| 5 Mr | 45 | 90.0 | 5 | 10.0 | 1.00 | 0.23 | 4.35 | 1.000 |
| 10 Mr | 50 | 100.0 | 0 | 0.0 | UD | UD | UD | **0.022** |
| 25 Mr | 50 | 100.0 | 0 | 0.0 | UD | UD | UD | **0.022** |
| Total | 190 | 95.0 | 10 | 5.0 |  |  |  |  |
| **Asao *B. pfeifferi* x Asao *S. mansoni*** | | | | |  |  |  |  |
| Negative control | 13 | 26.0 | 37 | 74.0 | 0.28 | 0.12 | 0.64 | **0.002** |
| 1 Mr | 28 | 56.0 | 22 | 44.0 | 1.00 |  |  |  |
| 5 Mr | 23 | 46.0 | 27 | 54.0 | 2.42 | 1.01 | 6.15 | **0.037** |
| 10 Mr | 31 | 62.0 | 19 | 38.0 | 4.64 | 1.83 | 11.98 | **<0.001** |
| 25 Mr | 30 | 60.0 | 20 | 40.0 | 4.27 | 1.69 | 10.96 | **0.001** |
| Total | 125 | 50.0 | 125 | 50.0 |  |  |  |  |
| **Asao *B. pfeifferi* x Mwea *S. mansoni*** | | | | |  |  |  |  |
| 1 Mr | 18 | 36.0 | 32 | 64.0 | 1.00 |  |  |  |
| 5 Mr | 36 | 72.0 | 14 | 28.0 | 4.57 | 1.81 | 11.70 | **<0.001** |
| 10 Mr | 26 | 52.0 | 24 | 48.0 | 1.93 | 0.80 | 4.65 | 0.107 |
| 25 Mr | 28 | 56.0 | 22 | 44.0 | 2.26 | 0.94 | 5.49 | **0.045** |
| Total | 108 | 54.0 | 92 | 46.0 |  |  |  |  |
| **Overall** |  |  |  |  |  |  |  |  |
| Negative control | 26 | 26.0 | 74 | 74.0 | 0.19 | 0.11 | 0.34 | **<0.001** |
| 1 Mr | 129 | 64.5 | 71 | 35.5 | 1.00 |  |  |  |
| 5 Mr | 141 | 70.5 | 59 | 29.5 | 1.32 | 0.85 | 2.05 | 0.200 |
| 10 Mr | 156 | 78.0 | 44 | 22.0 | 1.95 | 1.22 | 3.11 | **0.003** |
| 25 Mr | 151 | 75.5 | 49 | 24.5 | 1.70 | 1.08 | 2.68 | **0.016** |
| Total | 603 | 67.0 | 297 | 33.0 |  |  |  |  |

^¥^ - Odds Ratio; ^€^ - 95% Confidence Interval
